# Twenty Years of Full-Mouth Disinfection: The Past, the Present and the Future

**DOI:** 10.2174/1874210601812010435

**Published:** 2018-05-31

**Authors:** Ange Désiré Pockpa, Assem Soueidan, Pauline Louis, Nadin Thérèse Coulibaly, Zahi Badran, Xavier Struillou

**Affiliations:** 1 Clinical Investigation Unit 11 Odontology, CHU Nantes, Nantes, France; 2 Inserm, UMR 1229, RMeS, Regenerative Medicine and Skeleton, Université de Nantes, ONIRIS, Nantes, F-44042, France; 3 Department of Periodontology, Faculty of Dental Surgery, University of Felix Houphouët Boigny, Abidjan, Ivory Coast; 4 Department of Periodontology, Faculty of Dental Surgery, University of Nantes, Nantes, France; 5 Faculty of Dentistry, McGill University, Montreal, QC, Canada

**Keywords:** Periodontal disease, Periodontal treatment, Scaling and root planing, Full-mouth disinfection, Chlorhexidine, Antibiotics or probiotics

## Abstract

**Background:**

Conventional periodontal treatment, performed quadrant by quadrant in multiple visits, was re-evaluated in the early 1990s when the full-mouth disinfection concept was introduced. Over the years, several modifications to the full-mouth disinfection approach have been suggested.

**Objective:**

The purpose of this article is to review the evolution of full-mouth disinfection during the past 20 years, to specify its indications and to consider the prospects for this approach.

**Materials and Methods:**

An electronic and manual search of the literature, ending in December 2016, was performed by two independent researchers. Only pivotal studies and randomized controlled clinical trials published in the English language that evaluated a new approach to full-mouth disinfection were selected.

**Results:**

According to the studies included in our analysis (21 articles), several modified full-mouth disinfection protocols have been designed including: full-mouth treatment without chlorhexidine, the extension of hygiene methods and an increase in the duration of post-treatment chlorhexidine use, the replacement of chlorhexidine with other antiseptics, supplementation with antibiotics or probiotics, full-mouth antimicrobial photodynamic therapy and one-stage full-mouth disinfection combined with a periodontal dressing.

**Conclusion:**

Since 1995, several modifications have been suggested to improve the effectiveness of full-mouth disinfection. The majority of the studies demonstrate that the results obtained with full-mouth disinfection and its variants are equivalent to each other and to those obtained with the conventional quadrant method. Currently, the selection of this technique remains empirical and depends on the preferences of the practitioner and the patient. In the future, a patient-centered approach should be the best indication for the use of this technique.

## 1 INTRODUCTION

The mechanical treatment of Periodontal Disease (PD) involves scaling and root planing performed quadrant by quadrant in multiple visits spaced over one to two weeks (QSRP) [1]. This conventional PD treatment strategy was reevaluated in the early 1990s when the Full-Mouth Disinfection (FMD) concept was introduced. The principle of FMD is based on the scaling and root planning of all pockets and the treatment of all oral niches in two visits within 24 hours [2]. The aims of the FMD approach are twofold: first, to avoid the potential rapid translocation of periodontal pathogens; and second, to prevent the reinfection of previously treated sites by untreated pockets or by other intraoral niches [3, 4]. The original FMD protocol begins with motivating and instructing the patient in good oral hygiene techniques (Table **1**). The protocol proceeds as follows: a) scaling and root planing all teeth under local anesthesia during a 24 hour period spanning two consecutive days; b) brushing the back of the tongue with 1% Chlorhexidine (CHX) gel for a period of 1 minute; c) washing the mouth twice with 10 mL of 0.2% CHX for 1 minute, with gargling for the final 10 seconds; and d) performing the subgingival irrigation of all pockets with 1% CHX gel 3 times for 10 minutes each using a graduated syringe set at 6 and 8 mm immediately after each of the 2 sessions and 8 days later. At home, the patient is to comply with the recommendations of the dental practitioner (for 2 weeks, the patient is to wash the mouth twice daily with 10 mL of 0.2% CHX and use brushing aids) [5] (Table **1**). Since 1995, several modifications to the original FMD protocol have been suggested. The purpose of this article is to review the evolution of FMD during the past 20 years, to specify its indications and to consider the prospects for this approach.

## 2 MATERIALS AND METHODS

### 2.1 Review Question

How has the FMD protocol evolved during the past 20 years?

### 2.2 Search Strategy

This literature review was conducted by two independent reviewers (PZ and LP) using the PubMed/MEDLINE and Cochrane databases. Disagreements were resolved by discussion and third-party review (XS). The final search was performed in December 2016.

The following search terms were used: full mouth, disinfection, scaling and root planning, Quirynen, one-stage FMD, and periodontitis. Abstracts and the corresponding original articles were selected for review from the available titles. All articles that focused on the FMD concept were comprehensively reviewed. The bibliographies of the selected articles were subsequently reviewed to identify additional publications.

This electronic search was supplemented by a manual search of the following journals: Journal of Clinical Periodontology, Journal of Periodontology, Periodontology 2000, Clinical Oral Investigations, and Clinical Oral Implant Research.

### 2.3 Inclusion and Exclusion Criteria

Only Randomized Controlled Trials (RCTs) in which the original FMD protocol was modified were eligible for inclusion in this review. This study was limited to articles published in the English language.

Interventional studies, retrospective case-control studies, cross-sectional studies, case series, case reports, editorials, reviews, and animal studies were excluded from this review. In addition, studies that used the original FMD protocol and articles not published in English were excluded.

### 2.4 Data Extraction

The following data were extracted from the selected articles: names of authors, year of publication, number of patients, length of study, type of periodontitis treated, an amendment to the FMD protocol and main outcomes (Table **2**).

## 3 RESULTS

Since the FMD technique was first described, several teams have made changes to the protocol, and we have identified a total of 8 modified protocols: full-mouth treatment without CHX [6-9], the extension of hygiene methods and an increase in the duration of posttreatment CHX use [5], the replacement of CHX with other antiseptics [10-13], supplementation with antibiotics [14-22] or probiotics [21], full-mouth antimicrobial photodynamic therapy [23], and the most recent modification, one-stage FMD combined with a periodontal dressing [24] (Table **2**).

### 3.1 Evolution of the FMD Concept

#### 3.1.1 Full-Mouth Treatment without CHX

In 2000, Quirynen **et al**. proposed the removal of CHX use from the original protocol, thereby creating the full-mouth scaling approach (FMS). Quirynen **et al**. conducted a longitudinal study comparing FMS (test group 1) to FMD (test group 2) and Quadrant Scaling and Root Planing (QSRP) (control group) [6] and observed additional benefits in the two test groups in terms of pocket depth reduction (approximately 1.5 mm) and clinical attachment gain (approximately 2 mm). However, no statistically significant differences between the test groups were observed [6]. Additionally, motile microorganisms and spirochetes were significantly decreased in only the FMD group, and this difference lasted for up to 2 months posttreatment. However, this difference was not observed beyond 2 months [6]. In 2009, using a similar methodology, Swierkot **et al**. observed a greater reduction in pocket depths and gingival bleeding with the FMS protocol than that with the FMD protocol at 2 months. However, at 8 months, no significant difference was observed [8]. Apatzidou **et al*.* compared the FMS group to the QSRP group and observed that patients treated with FMS had more postoperative pain compared to those who received conventional therapy with CHX [7]. In 2013, Santos **et al**. investigated the treatment of chronic periodontitis in patients with type II diabetes (FMD compared with FMS + placebo) and observed no significant clinical differences between the results of these treatments for a posttreatment period of up to 12 months [9].

#### 3.1.2 Extension of Hygiene Methods and Increased Duration of Posttreatment CHX Use

Bollen **et al**. assessed the use of CHX (mouthwashes and tonsil sprays) for a period of 2 months after treatment instead of 2 weeks [5]. These investigators compared FMD with 2 months of CHX treatment (test group) to QSRP (control group) by evaluating the clinical and microbiological effects of these treatments after 2 and 4 months. Samples of saliva and gingival, lingual, and mucosal plaques were collected. At 2 and 4 months, Bollen **et al**. observed significantly higher clinical attachment gains in the test group than those in the control group (1.5 mm versus 0.3 mm in deep pockets; 0.9 mm versus 0.1 mm in pockets of moderate depth). In terms of the microbiological effect, they noted a significant decrease in *Porphyromonas gingivalis* (Pg), *Prevotella intermedia* (Pi), and spirochetes in the test group. However, at the end of this study, the authors could not demonstrate a direct relationship between the observed results and the increased CHX use. According to the authors, these results could be due to the effectiveness of the full-mouth method compared with that of the quadrant method [5].

#### 3.1.3 Replacement of CHX with other Types of Antiseptics

In 2006, Quirynen **et al**. considered the possibility of using Amine Fluoride/stannous fluoride (AF) in the original protocol to complement or to substitute for CHX [10]. This study compared these two regimens to the conventional quadrant method. At 8 months posttreatment, no additional benefit was observed with the use of AF either alone or combined with CHX [10]. Using a similar methodology, Wang *et al*. studied the possibility of using povidone-iodine (Betadine^®^) in the FMD protocol [11] by comparing QSRP (control) to a modified FMD protocol including an irrigation treatment with either water (test 1) or povidone-iodine (test 2). Blood samples were taken before treatment and at 1, 3 and 6 months after treatment. The study aimed to compare the expression of serum antibodies in response to the following periodontal pathogens: Pg, *Aggregatibacter actinomycetemcomitans* (Aa), and *Treponema denticola* (Td). Compared to the control group, both test groups showed significant reductions in anti-Pg and anti-Aa antibodies at 1 and 3 months. These authors suggested that povidone-iodine could be a reliable alternative to CHX in the FMD protocol [11]. A few years later, in a study investigating the use of essential oils as an adjuvant to or substitute for CHX [12, 13], the authors reported that essential oils were beneficial for the reduction of pocket depth and plaque and gingival indices [12, 13]. However, the results of the microbiological analysis were less clear.

#### 3.1.4 Supplementation with Antibiotics

The hypothesized benefit of adding antibiotics to the FMD protocol has been the subject of several studies [14-22]. In 2007, Gomi **et al**. compared the QSRP protocol (control group) an FMD protocol with Azithromycin (AZT) added (test group) [14]. AZT was administered during the three days preceding the mechanical treatment. The clinical and microbiological parameters were recorded over a 6 month period, and an improvement in the clinical parameters at 2 and 6 months posttreatment was observed in the AZT group [14]. At 2 months, the elimination of periopathogenic bacteria was significantly greater in the test group than that in the control group [14]. The authors concluded their study by claiming that the addition of AZT to the FMD protocol was clinically and microbiologically effective [14]. Similar observations were noted by Yashima **et al**. [15]. However, recently, Fonseca **et al**. showed that the addition of AZT did not provide additional clinical benefits compared to the FMD technique alone [22]. In this study, the authors divided the samples into 6 groups and compared different protocols: (a) a full-mouth approach without CHX (FMS), (b) FMD alone, (c) FMD + AZT, d) QSRP without CHX, e) QSRP + CHX, and f) QSRP + AZT. At 3 months, a significant reduction in the depth of deep pockets, gingival inflammation, plaque index, and clinical attachment gain was observed in each group [22]. Compared to the other groups, the group receiving FMD alone exhibited a greater reduction in pocket depth and a lower rate of PD at 6 months [22]. Cionca **et al**. investigated the addition of Amoxicillin (Amox) and Metronidazole (MTZ) to the FMD protocol using a regimen of 375 mg of Amox and 500 mg of MTZ three times a day for 7 days [16, 17]. At 6 months, Cionca **et al**. observed a greater reduction in the depth of deep pockets in the test group than that in the control group [16]. Moreover, the test group had a smaller number of residual pockets of more than 4 mm in depth than the control group (*p* = 0.005) and had a significantly reduced need for complementary surgical treatment [16]. However, beyond 6 months, no significant differences in these clinical parameters were observed [16]. In terms of the microbiological effect, Cionca *et al*. observed the elimination of Aa in the test group but not in the control group at 3 months posttreatment. Additionally, lower levels of Pg (*p* = 0.013) and *Tannerella forsythia* (Tf) (*p* = 0.007) were observed in the test group than those in the control group [17]. However, these results were not confirmed at 6 months [17]. Similarly, Varela **et al**. reported that, at 3 months, an additional clinical benefit in the treatment of aggressive periodontitis was observed with the addition of Amox and MTZ to the FMD protocol (500 mg amoxicillin + 250 mg metronidazole, three times a day for 10 days) [18]. However, according to a similar study by Aimetti **et al**., the microbiological effects of the addition of Amox and MTZ remained for up to 6 months [19]. Preus **et al**. evaluated the efficacy of the addition of MTZ monotherapy to the FMD protocol [20]. They compared 4 protocols: a) FMD + 400 mg MTZ (three times a day for 10 days), b) FMD + placebo, c) QSRP + 400 mg MTZ (three times a day for 10 days), and d) QSRP + placebo. They reported that the addition of MTZ increased clinical attachment gains and reduced pocket depth [20]. However, at 12 months, FMD either with or without MTZ did not improve the clinical conditions beyond those obtained by conventional therapy [20].

#### 3.1.5 Addition of Probiotics

The addition of probiotics (*Lactobacillus reuteri* (LR) in tablet form) to the FMD protocol has also been considered [21]. Teughels **et al**. compared FMD with the twice daily administration of LR for 12 weeks (test group) to FMD with a placebo (control group). At 12 weeks, the authors observed a significant improvement in clinical and microbiological parameters, including a significant improvement in pocket depth and clinical attachment gain and a reduction in the periopathogenic bacterial load. They concluded that the oral administration of probiotic LR tablets in addition to scaling and surfacing by a comprehensive disinfection method would be useful in the treatment of chronic periodontitis [21].

#### 3.1.6 Full-mouth Antimicrobial Photodynamic Therapy

Sigush *et al*. conducted a study to evaluate the efficacy of dynamic phototherapy in addition to FMD on the eradication of *Fusobacterium nucleatum* (Fn) [23]. Patients received either FMD with a photosensitive solution that was activated by a laser (test group) or FMD with the unactivated photosensitive solution (control group). Compared to the control group at 3 months posttreatment, the patients in the test group had a greater reduction in pocket depth, better clinical attachment, and a significant reduction in Fn load [23].

#### 3.1.7 FMD Combined with a Periodontal Dressing

Keestra **et al**. evaluated the effects of adding the use of a periodontal dressing (Coe-Pak^®^ type) to the FMD protocol [24]. This approach resulted in a greater reduction in shallow and moderate-depth periodontal pockets. However, only deep pockets showed a tendency for improvement. According to the authors, this technique would provide additional short-term clinical benefit and would reduce postoperative pain [24].

## 4 DISCUSSION

The FMD concept has generated great enthusiasm over the last 20 years due to its many offered advantages. Indeed, FMD reduces both the number of sessions in the dental chair and the duration of the periodontal treatment. The shorter working time limits the risk of intraoral cross-contamination between treated and untreated sites [4], thereby allowing better control of the transmission of periodontopathogens between the bacterial niches. In addition, the FMD procedure is more comfortable and more economical for the patient and the practitioner. Since 1995, several modifications have been suggested to improve the effectiveness of FMD. These modifications include full-mouth treatment without CHX [6-9], the extension of hygiene methods and an increase in the duration of posttreatment CHX use [5], the replacement of CHX with other antiseptics [10-13], the addition of antibiotics [14-22] or probiotics [21], the use of photodynamic therapy [23], and the use of a periodontal dressing [24]. FMD without CHX reduced the outcomes of the clinical results, suggesting an important but prudently selected use for the protocol [7]. The use of CHX for longer than 15 days is unnecessary because of the undesirable side effects that are normally associated with prolonged CHX exposure. Similarly, a beneficial clinical effect, especially in the depth of deep periodontal pockets, is obtained when antibiotics are added to the FMD protocol [14-22]. However, there is no consensus on the value of supplementing FMD with antibiotic therapy in the treatment of severe chronic periodontitis. The other modifications have shown very good results, although their increased effectiveness does not reach significance when compared to the conventional treatment. According to the articles included in this review, the results obtained with FMD and its variants are not maintained over the long term; the results of the FMD approach and its variants are equivalent to those of the conventional quadrant approach [25]. Additionally, with FMD, the number of sessions is reduced, but the sessions are longer and more tiring. Thus, the criteria for choosing the FMD treatment method will depend on the habits and experience of the practitioner, the management of the planning phase and appointments of the practice, and patient availability, compliance, and preference.

## CONCLUSION AND PERSPECTIVES

At the end of the 1990s, the FMD concept was considered the best approach for periodontal treatment to avoid the reinfection of the already treated periodontal pockets [3, 4]. Over the years, several modifications to improve the effectiveness of FMD have been suggested. The majority of the studies have demonstrated that the results obtained with FMD and its variants are equivalent to those obtained with the conventional quadrant method. The FMD concept is based on a plausible scientific rationale. Currently, the selection of this technique remains empirical and depends on the preferences of the practitioner and the patient. Indeed, the selection of the FMD technique cannot be based on its greater effectiveness since the majority of the clinical studies have shown it is possible to obtain results equivalent to those obtained with the conventional method with any variant of the FMD technique.

The future of this technique will depend on the progress of research in personalized medicine, microbiology and inflammation. The diversity in the genetic, infectious and immunologic subtypes of periodontal disease argues in favor of personalized therapy. A better knowledge of the oral microbiota and the host response may allow greater precision in defining the indications for FMD. The analysis of the bacterial load, the bacterial composition, and the quality of the inflammatory response will facilitate the design of clinical studies to determine the clinical situations in which this technique could be beneficial. On the other hand, periodontal classification is based almost exclusively on clinical characteristics and offers very limited therapeutic guidance and little evidence of actually improving periodontal care.

In the classification of periodontal disease, it would be interesting to include parameters based on the nature of the periodontal pathology and the general state of health of the patient; such parameters could guide therapeutic choices, for example, the indication for antibiotic therapy as an addition to FMD.

Finally, the concurrent delivery of high-quality periodontal health care and reduction in treatment cost is an obvious challenge, but the FMD technique, which makes it possible to optimize the duration of treatment, could contribute to a reduction in treatment cost and could thus facilitate patient care.

## Figures and Tables

**Table 1:** Initial protocol of FMD (1)

1-SRP "full-mouth"	All teeth in two visits within 24 hours,under local anesthesia
2-Brushing the back of the tongue	For 1 minute, with 1% CHX gel
3-Mouthwash	2 times, for 1 minute, with 10mL of CHX at 0.2% and gargling the last 10 seconds to reach the tonsils
4-Subgingival irrigation of all pockets	3 times, for 10 minutes, with 1% CHX gel, after each of the 2 sessions, and repeated at D8, using a 6 and 8mm syringe labeled
5-Mouthwash (at home)	With 10mL of CHX at 0.2%, twice a day for 1 minute, over 2 weeks
6-Oral hygiene instructions	Tooth brushing, interdental cleaning with brushes or other hygiene aid, brushing of the tongue

**Table 2:** Characteristics of randomized controlled trial included in review.

** Author** ** (year) **	**Subjects**	**Type of PD**	**Amendment**	**Outcomes**
Quirynen *et al*. (1995) [2]	10	SCP	Initial Protocol (FMS + CHX _2wks_)	For deep pockets : more reduction of PPD in T group / C group (*p* < 0.05).
Bollen *et al*. (1998) [5]	16	SCP	FMS with CHX _2mths_	better results than in initial FMD protocol.
Quirynen *et al*. (2000) [6]	36	SCP	FMD without CHX	FMS alone > QRSP: more reduction of PPD and CAL gain (*p*<0.001; *p* <0.001).
Apatzidou *et al*. (2004) [7]	40	SCP	FMD without CHX	Both therapies (FMS alone and QRSP) improved clinical indices.
Quirynen *et al*. (2006) [10]	71	MCP	FMD with AF	Less reduction of PPD with AF alone ( FMS + AF < FMS + CHX or / FMS + CHX +AF *p*<0.05) .
Wang *et al*. (2006) [11]	36	CP	FMD with PId	Less reduction of Pg and Aa with Id versus Water or QRSP (*p*<0.005;**P**<0.005).
Gomi *et al*. (2007) [14]	34	SCP	FMD + AZT alone	Significant improvement of clinical parameters with AZT (PPD, BOP, GCF) (*P*<0.001).
Cavalca al. (2009) [12]	50	MCP	FMD with EO	Efficacy of EO to reduce PPD, PI and BOP.
Cortelli *et al*. (2009) [13]	50	MCP	FMD with EO	At 2 and 6 months, no significance difference between EO and placebo regarding microbiology parameters.
Yashima *et al*. (2009) [15]	30	CP	FMD + AZT alone	Significant improvement of clinical parameters with AZT (*P*<0.01; *P*<0.05).
Cionca *et al*. (2009) [16]	47	CP	FMD + Amox & MTZ	At 6 months, less sites with PDP > 4 mm with Amox +MTZ versus placebo.
Swierkot *et al*. (2009) [8]	25	CP	FMD without CHX	At 1 and 2 months, more reduction of PPD, BOP in FMD with or without CHX versus QRSP alone.
Cionca *et al*. (2010) [17]	51	CP	FMD + Amox & MTZ	Significant reduction of Pg (*P* = 0,013) and Tf (*P* = 0,007) with FMD combined to Amox&MTZ compared to placebo or FMD alone.
Sigusch *et al*. (2010) [23]	24	CP	FMD with PTD	Better improvements of PPD and CAL gain with FMD combined to PTD versus FMD alone.
Varela *et al*. (2011) [18]	25	AP	FMD + Amox & MTZ	Better clinical improvements (PPD, CAL) on sites with PDi <5mm (*p* <0.03) using FMD combined to Amox&MTZ versus FMD alone.
Aimetti *et al*. (2012) [19]	39	AP	FMD + Amox & MTZ	Up to 6 months, better reduction of Aa + red complexes in site with PDi > 5 mm using FMD combined to Amox&MTZ versus FMD alone .
Preus *et al*. (2013) [20]	184	MCP & SCP	FMD + MTZ alone	Up to 12 months in groups with MTZ, sites with PDi > 5 mm obtained better PPD reduction + better CAL gain.
Santos *et al*. (2013) [9]	38	CP	FMD without CHX	Up to 12 months similar clinical improvement with or without CHX.
Teughels *et al*. (2013) [21]	30	CP	FMD + PRB_12 wks _	Reduction in surgical treatment need with PRB.
Keestra *et al*. (2014) [24]	24	CP	FMD + periodontal dressing	Reduction of postoperative pain with adjunction of periodontal dressing .
Fonseca *et al*. (2015) [22]	85	CP	FMD + AZT	Addition of AZT did not provide additional clinical benefit compared to the FMD technique alone.

**+**: associated to; &: and ; **Amox** :Amoxicillin ; **AZT** :Azithromycin; **AF**:Amine Fluoride; **AP** :Agressive Periodontitis; **BOP**, Bleeding On Probing ; **CAL**, Clinical Attachment Level; **CHX**:Chlorhexidine; **CHX _2months_** : Chlorhexidine during 2 months; **CHX _2wks_** : Chlorhexidine during 2 weeks ; **CP** : Chronic Periodontitis ; **EO** :Essential Oils ; **FMD** : Full Mouth Disinfection ; **FMS** : Full Mouth Scaling; **GCF** :Gingival Crevicular Fluid. **H2O**: water; **MCP**: Moderate Chronic Periodontitis; **MTZ**: Metronidazole; **PD**:Periodontitis ; **PDi **: Initial Probing Depth; **Pg **:Porphyrhomonas Gingivalis ; **PId** Povidone Iodine **PI**, Plaque Index; **PPD**, Probing Pocket Depth; **PRB_12 wks_**_ :_Probiotics During 12 weeks;**PTD**:Photodynamic Therapy; **QSRP**:Conventional Treatment by Quadrant ;**RCT**: Randomized Control Trial ;**SCP**:Severe Chronic Periodontitis
